# The Design and Analysis of a Novel Split-H-Shaped Metamaterial for Multi-Band Microwave Applications

**DOI:** 10.3390/ma7074994

**Published:** 2014-07-02

**Authors:** Sikder Sunbeam Islam, Mohammad Rashed Iqbal Faruque, Mohammad Tariqul Islam

**Affiliations:** 1 Centre for Space Science, Research Centre Building, Universiti Kebangsaan Malaysia, Bangi, Selangor D.E. 43600, Malaysia; E-Mail: rashed@ukm.my; 2 Department of Electrical, Electronic and Systems Engineering, Faculty of Engineering and Built Environment, Universiti Kebangsaan Malaysia, Bangi, Selangor D.E. 43600, Malaysia; E-Mail: tariqul@ukm.my

**Keywords:** double-negative (DNG) material, left-handed material (LHM), multi-band

## Abstract

This paper presents the design and analysis of a novel split-H-shaped metamaterial unit cell structure that is applicable in a multi-band frequency range and that exhibits negative permeability and permittivity in those frequency bands. In the basic design, the separate split-square resonators are joined by a metal link to form an H-shaped unit structure. Moreover, an analysis and a comparison of the 1 × 1 array and 2 × 2 array structures and the 1 × 1 and 2 × 2 unit cell configurations were performed. All of these configurations demonstrate multi-band operating frequencies (S-band, C-band, X-band and K_u_-band) with double-negative characteristics. The equivalent circuit model and measured result for each unit cell are presented to validate the resonant behavior. The commercially available finite-difference time-domain (FDTD)-based simulation software, Computer Simulation Technology (CST) Microwave Studio, was used to obtain the reflection and transmission parameters of each unit cell. This is a novel and promising design in the electromagnetic paradigm for its simplicity, scalability, double-negative characteristics and multi-band operation.

## 1. Introduction

A metamaterial can be defined as an artificial electromagnetic structure that may have, in a specific frequency range, certain exotic electromagnetic properties that are normally not found in nature. Such artificial materials have ushered in a new era in modern science. They possess extraordinary electromagnetic properties, such as the ability to exhibit negative values of permittivity and permeability, simultaneously, in a specific frequency range. In 1968, Victor Veselago first noted that materials with simultaneous negative permeability (μ < 0) and permittivity (ε < 0) displayed certain unique properties compared to ordinary materials that are found in nature [1], but until 1999, there was little exploration of this phenomenon, because of the lack of the availability of such natural materials. In 2000, Smith *et al.* [2] successfully demonstrated a new artificial material with this exotic property (*i.e.*, both μ and ε were negative), which is defined as a left-handed metamaterial. Metamaterials that exhibit both negative permittivity and negative permeability simultaneously are also called double-negative (DNG) metamaterials. Metamaterials can also be single negative materials, either epsilon negative (ENG) or mu-negative (MNG) materials. Metamaterials, with single- or double-negative properties, can be used to achieve superior performance in many important applications, such as SAR reduction [3,4,5,6], antenna design [7,8,9], invisibility cloaking [10,11], polarization rotators [12], filters and other applications [13,14,15,16,17,18,19,20]. Recently, in the communication sector, especially in high-gain multi-band antenna design, multi-band metamaterial or an array of metamaterials with negative refractive indices or near-zero refractive indices have become a promising approach for researchers, although very few studies focused on such designs can be found in the literature [21,22]. Multi-band metamaterial absorbers also have some promising applications in explosives detection, bolometers and thermal detectors [23]. Various alphabetic metamaterial structures have been proposed for specific applications; for example, Hayet Benosman *et al.* [24,25] have proposed a double “S-shaped” metamaterial in the microwave range. Their metamaterial exhibits a negative refractive index between 15.67 and 17.43 GHz. Few studies have yet been conducted based on their design. Anik Mallik *et al.* [26] have proposed a rectangular “U-shaped” metamaterial that exhibits double-negative characteristics at approximately 5, 6 and 11 GHz for different array configurations. Evren Ekmekçi *et al.* have demonstrated a “V-Shaped” metamaterial. This structure exhibits double-negative characteristics at only 8.10 GHz [27]. Abdallah Dhouibi *et al.* [28] have presented a “Z-shaped” metamaterial that is applicable at 4.5 GHz. However, their design has been proven for single-negative (SNG) characteristics with negative permittivity only. 

In this paper, a new H-shaped metamaterial unit cell structure is proposed, which contains two joined split-square resonators and exhibits resonance frequency in the multi-band (S-band, C-band, X-band and K_u_-band) range of the microwave spectra [29]. It also exhibits double-negative properties at those frequencies. The commercially available electromagnetic simulation software package, Computer Simulation Technology (CST) Microwave Studio, was used to obtain the reflection and transmission parameters of the unit cell and to monitor the resonance frequencies. These parameters can be used to determine the effective permeability (μ) and permittivity (ε) for the proposed configurations.

## 2. Materials and Methods

**Figure 1:**
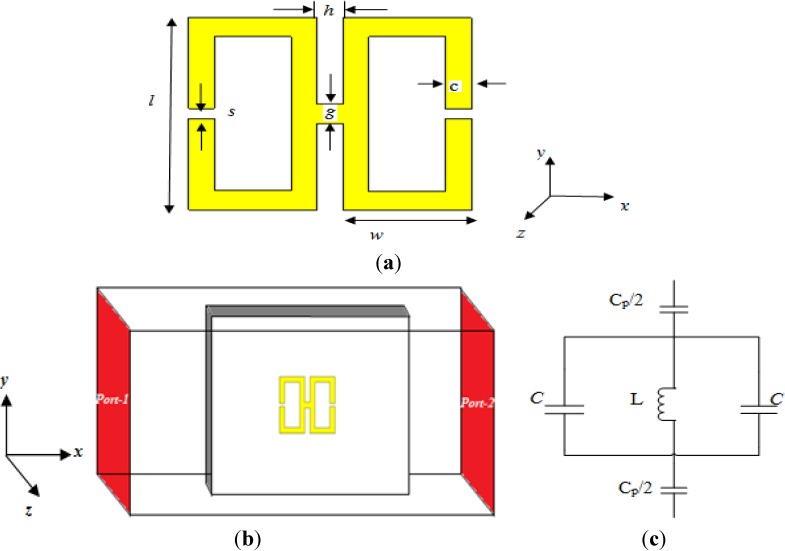
(**a**) The proposed H-shaped unit cell structure; (**b**) simulation geometry; (**c**) equivalent circuit of the unit cell.

A schematic view and the design parameters of the proposed double-negative unit cell structure are presented in Figure 1a. The design of the structure is based on two copper split-square resonators. All elements have the same thickness of 0.035 mm. The width of each split-square resonator is 1 mm, and the outer length is 10 mm, with a 0.5-mm split. The gap between the two resonators is 1 mm, and they are joined to form an H-shape by a copper line of 1 mm in width. The structure is printed on a square-shaped FR-4 substrate with a dielectric constant of ε_r_ = 4.3, a dielectric loss-tangent of tanδ_ε_ = 0.025, a side length (a) and width (b) of *a* = *b* = 30 mm and a thickness of *t* = 1.6 mm. The design specifications for the unit cell are provided in Table 1. 

**Table 1:** Design specifications of the unit cell.

The unit cell parameters	Value (mm)
*c*	1
*g*	1
*h*	1
*l*	10
*s*	0.5
*w*	5

In this study, a finite-difference time-domain (FDTD) study based on Computer Simulation Technology (CST) Microwave Studio was used to calculate the S-parameters using 100 frequency samples. The structure to be tested was placed between two waveguide ports at positive and negative locations on the *x*-axis and excited by an electromagnetic wave in the direction of the *x*-axis. The perfect electric conductor (PEC) boundary condition was applied to the *y-*axis, and the *z-*axis was defined as a perfect magnetic conductor (PMC) boundary; the simulation geometry is displayed in Figure 1b. A frequency-domain solver was used for the simulation. The normalized impedance was set to 50 ohms. The simulation was performed for the frequency range of 2–15 GHz. 

**Figure 2:**
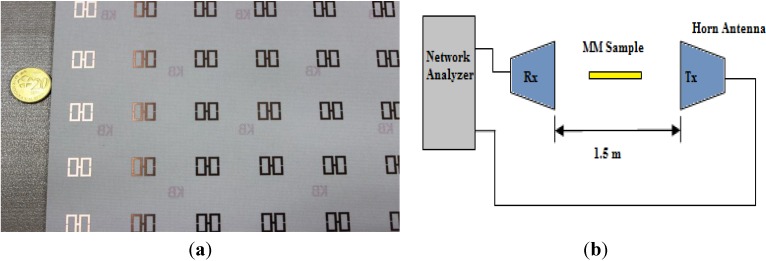
(**a**) The prototype array of the H-shaped structure; (**b**) experimental setup.

A prototype was fabricated for measurement, as shown in Figure 2a. The dimensions of the prototype are 240 × 240 mm^2^, and it contains 8 × 8 unit cells of the same materials described in the simulation procedure. The measurements were acquired in a semi-anechoic chamber with two 1–18 GHz broadband horn antennas placed 1.5 m apart. The prototype was placed between the horn antennas in the same plane, analogous to the simulation geometry, to allow the wave to propagate over the prototype. Moreover, for calibration purposes, transmittance measurements were acquired both with and without the prototype in position. An Agilent E8363D vector network analyzer was used to calculate the transmission co-efficient. The experimental setup is shown in Figure 2b.

## 3. Equivalent Circuit Model

In essence, this type of metamaterial structure consists of passive *LC* circuits with a resonance frequency of:
*f* = 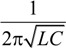
(1)
where *L* and *C* are the total inductance and capacitance, respectively, of the structure. In the above structure, the inductances are formed by the metal loops and the capacitances are formed by the gaps. When the applied electromagnetic wave propagates along the structure, the coupling between the gaps and the electric fields produces electric resonances, and the magnetic resonances are formed by the coupling between the magnetic fields and the loops. Normally, according to the quasi-static theory, the total capacitance formed between gaps is:
*C* = 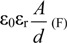
(2)
where ε_0_ and ε_r_ are the permittivity of free space and the relative permittivity, respectively; “*A*” is the cross-sectional area of the gap; and “*d*” is the gap length, which, for our design, is “s”. However, to determine the inductance, both the internal and external inductance must be considered for the proposed structure. The equivalent circuit is illustrated in Figure 1c, where *L* and *C* represent the inductance and capacitance, respectively, as passive elements, and *C*_p_ is the capacitance required for the *LC* resonance that forms between two consecutive unit cells. The resonance frequency for the proposed H-shaped resonator is:
*f* = 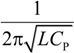
(3)
where the inductance for the proposed structure can be approximated as:
*L* ≈ 
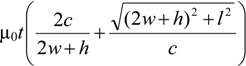
(4)
and the equivalent capacitance can be approximated as:
*C*_p_ ≈ 
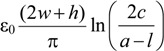
(5)
where the free-space permeability is μ_0_ = 4π × 10^−7^ H/m and the free-space permittivity is ε_0_ = 8.85 × 10^−12^ F/m.

A more detailed discussion of the above equations can be found in [30,31,32].

## 4. Results and Discussion

**Figure 3:**
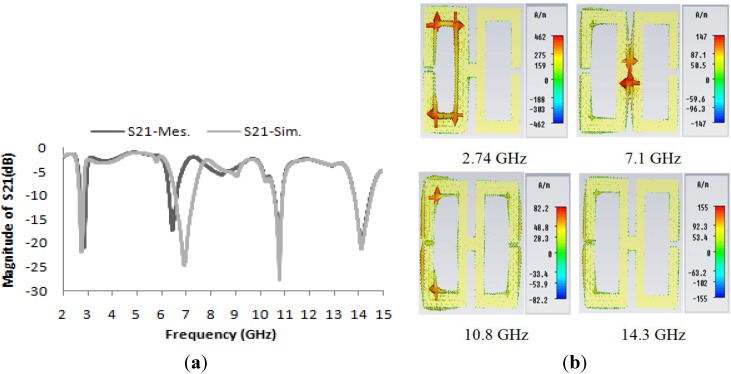
(**a**) Magnitudes of the measured and simulated transmission parameters (S_21_) in units of dB for the unit cell; (**b**) current distributions in the unit cell structure at various frequencies.

The transmission coefficient (S_21_) of the unit cell and the unit cell current distribution are presented in Figure 3a,b, respectively. In Figure 3a, the simulated and measured spectra for the transmission characteristics S_21_ of the proposed unit cell structure reveal that the simulated magnitude of S_21_ exhibits four resonances in four different microwave bands at frequencies of 2.74 GHz, 7.122 GHz, 10.855 GHz and 14.337 GHz and that the experimental result for S_21_ agrees well with the simulation, except for the slight shift in the second resonance to 6.446 GHz instead of 7.122 GHz. Nevertheless, the C-Band is maintained. This shift most likely occurred because of a fabrication error. However, according to the circuit model for the basic H-shaped unit cell, the calculated resonance frequency is 10.9 GHz, which is in very good agreement with the simulated value of 10.85 GHz. Figure 3b shows the current distributions at various resonance frequencies in the unit cell structure.

**Figure 4:**
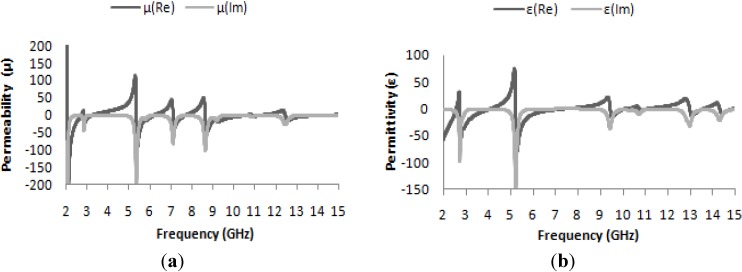
(**a**) Real and imaginary values of effective permeability (μ) *vs.* frequency; (**b**) real and imaginary values of effective permittivity (ε) *vs.* frequency.

The effective permeability and permittivity of the medium can be determined from the simulated complex S_21_ and S_11_ parameters using the method described in [33]. These results are presented in Figure 4a,b; the effective permeability as a function of frequency is presented in Figure 4a, and similarly, the effective permittivity is presented in Figure 3b. In both Figure 4a,b, it is clearly apparent that at the resonance frequencies of 2.74 GHz, 7.122 GHz, 10.853 GHz and 14.337 GHz, both the permeability and permittivity take on negative values; these values are listed in Table 2. Thus, the structure can be said to be a DNG metamaterial. 

**Table 2:** Real values of ε, μ and η at the desired resonance frequencies for the unit cell.

Frequencies (GHz)	Value of ε	Value of μ	Value of η
2.74	−35.6	−0.87	−5.7
7.122	−1.4	−3.2	−7
10.855	−3.3	−0.35	−1.9
14.337	−1.9	−0.84	−2.64

Normally, a charge builds up in the gap of a split-ring resonator if it is subjected to a varying magnetic field. At a low frequency, the current of the oscillator remains in the phase of the applied field, but at a higher frequency, the current begins to lag and fails to remain in phase with the applied field, thus producing negative permeability at that frequency. 

**Figure 5:**
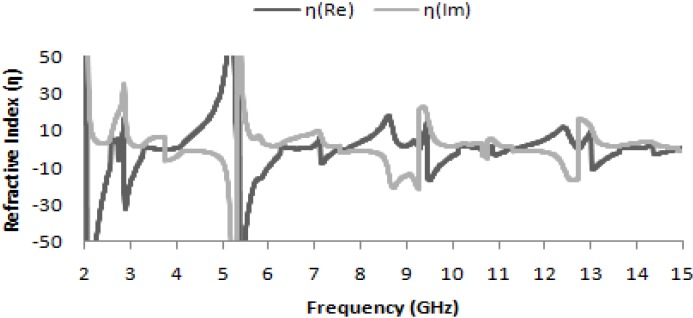
Real and imaginary values of refractive index (η) *vs.* frequency.

Accordingly, in Figure 5, where the real part of the refractive-index curve is plotted as a function of frequency, the refractive indices are also found to be negative for frequencies of 2.74 GHz, 7.122 GHz, 10.853 GHz and 14.337 GHz; the corresponding values are presented in Table 2. Thus, the manifestation of negative permeability over these frequency bands may also be useful in long-distance communication applications. 

### 4.1. Design and Analysis of a 1 × 1 Array of the Unit Structure

**Figure 6:**
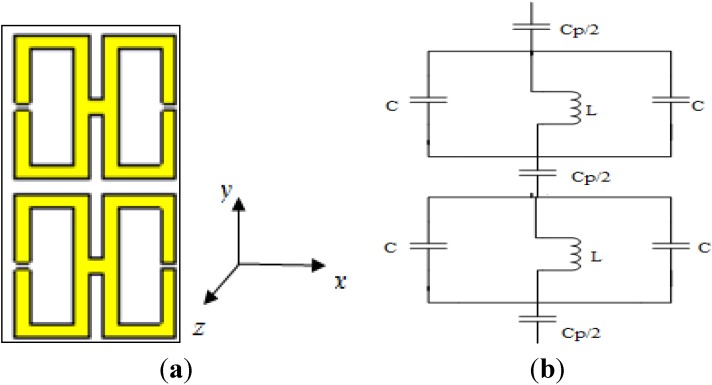
(**a**) The 1 × 1 array of the H-shaped unit structure; (**b**) the equivalent circuit of the 1 × 1 array of the H-shaped unit structure.

Figure 6a illustrates a 1 × 1 array of the unit structure formed vertically on the same substrate on which the basic unit structure was designed. The equivalent circuit of it is seen in Figure 6b. The distance between the two units is 1 mm. Operating frequencies between 2 and 15 GHz were considered, and the same methodology as used previously was used to evaluate the performance of this array. 

**Figure 7:**
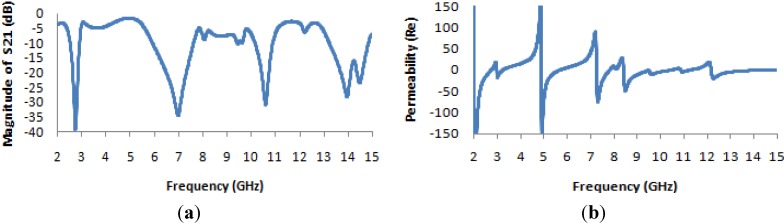
(**a**) The magnitude of the transmission parameter (S_21_) in units of dB for the 1 × 1 array of the unit structure; (**b**) the real value of permeability *vs.* frequency for the 1 × 1 array of the unit structure.

**Figure 8:**
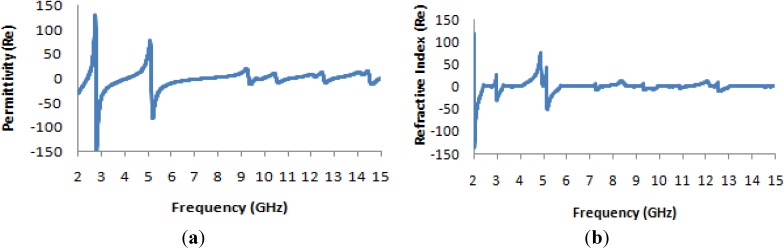
(**a**) The real value of permittivity *vs.* the frequency for the 1 × 1 array of the unit structure; (**b**) the real value of the refractive index *vs.* frequency for the 1 × 1 array of the unit structure.

From Figure 7a, it is apparent that the transmission coefficient has resonances at the same points as for the previously considered structure (the unit cell), but with strongly negative magnitudes. Figure 7b and Figure 8a present the real values of the effective permeability and permittivity *vs.* frequency for the 1 × 1 array of the unit structure.

In Figure 8a, it is evident that the highest magnitude of the permittivity has shifted to lie between 2 and 3 GHz, whereas in the case of the unit cell, it lies between 5 and 6 GHz. Meanwhile, the highest magnitude of the permeability for the unit cell arises midway between 5 and 6 GHz, as seen in Figure 4a, but in the case of the 1 × 1 array, it arises at a frequency of approximately 5 GHz, as seen in Figure 7b. Moreover, it is also observed that the real values of the permittivity and permeability are negative in the desired frequency bands; these values are summarized in Table 3. In Figure 8b, the behavior of the refractive index as a function of frequency is presented, and this quantity also takes on negative values in the multi-band range.

**Table 3:** Real values of ε, μ, and η in the desired resonance frequency bands for the 1 × 1 array of the unit structure.

Frequencies (GHz)	Value of ε	Value of μ	Value of η
2.75	−10.64	−0.21	−1.8
7.26	−0.60	−27.2	−6.73
10.855	−2.80	−0.67	−2.88
14.519	−2.55	−0.14	−0.88

### 4.2. Design and Analysis of a 1 × 1 Unit Cell

**Figure 9:**
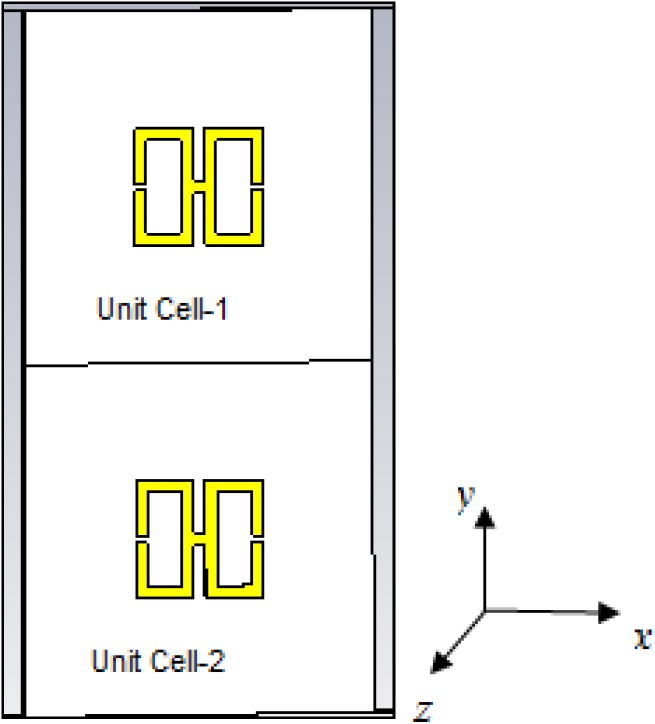
The 1 × 1 H-shaped unit cell.

The design of the 1 × 1 unit cell is illustrated in Figure 9, where it can be seen that the entire unit cell is vertically arranged. Operating frequencies between 2 and 15 GHz were considered, and the same methodology as used previously was used to evaluate the performance of the cell. 

**Figure 10:**
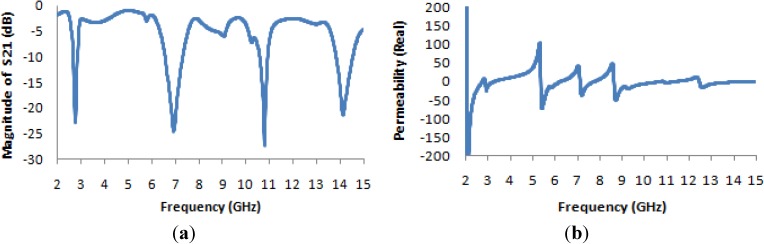
(**a**) The magnitude of the transmission parameter (S_21_) in units of dB for the 1 × 1 unit cell; (**b**) the real value of permeability *vs.* the frequency for the 1 × 1 unit cell.

**Figure 11:**
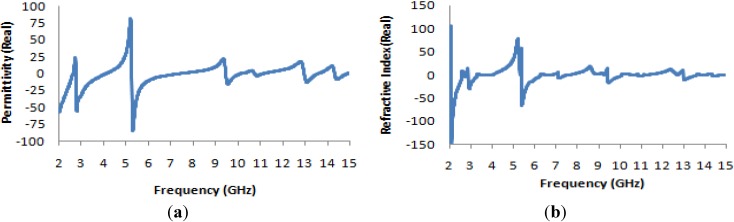
(**a**) The real value of permittivity *vs.* the frequency for the 1 × 1 unit cell; (**b**) the real value of the refractive index *vs.* frequency for the 1 × 1 unit cell.

Figure 10 presents the S-parameter and permeability curves for the 1 × 1 unit cell. From Figure 10a, it is clearly evident that the transmission co-efficient exhibits the same characteristics as in the case of the single unit cell. The permeability curve presented in Figure 10b and the permittivity curve presented in Figure 11a also exhibit the same characteristics as in the case of the single unit cell, as does the refractive-index curve presented in Figure 11b. 

Thus, it is demonstrated that for two separate unit cells, the effective parameters are identical to those of a single unit cell and clearly differ from those of the two-unit structure in the same substrate plane. 

It is notable that in the lowest frequency band (the S-band), the permittivity and the refractive index vary slightly from the case of the unit cell, but at higher frequencies, these quantities are nearly identical in both cases. On the other hand, the permeability varies slightly between the two cases at both the S- and C-band frequency levels, as seen in Table 4. However, from Table 4, it is also clear that for 1 × 1 unit cell operation, in all four frequency bands (the S-, C-, X- and K*_u_*-bands), the metamaterials exhibit double-negative characteristics. 

**Table 4:** Real values of ε, μ and η in the desired resonance frequency bands for the 1 × 1 unit cell.

Frequencies (GHz)	Value of ε	Value of μ	Value of η
2.74	−11.51	−1.19	−21.64
7.135	−1.32	−12.60	−7.17
10.855	−3.27	−0.19	−1.78
14.337	−1.17	−0.85	−2.58

### 4.3. Design and Analysis of a 2 × 2 Array of the Unit Structure

**Figure 12:**
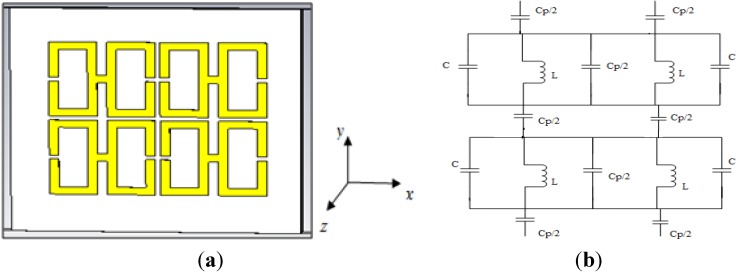
(**a**) The 2 × 2 array of the H-shaped unit structure; (**b**) the equivalent circuit of the 2 × 2 array of the H-shaped unit structure.

The 2 × 2 array of the unit structure and the equivalent circuit is illustrated in Figure 12a,b, respectively. It is formed in a vertically stacked and side-by-side form on the same substrate on which the basic unit structure was designed. The distance between two units is 0.5 mm on each side. Operating frequencies between 2 and 15 GHz were considered, and the same methodology as used previously was used to evaluate the performance of this array.

**Figure 13:**
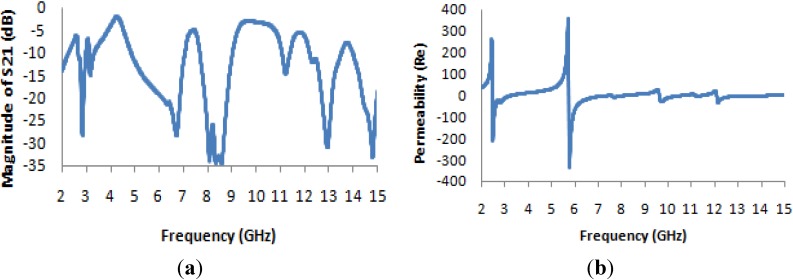
(**a**) The magnitude of the transmission parameter (S_21_) in units of dB for the 2 × 2 array of the unit structure; (**b**) the real value of permeability *vs.* the frequency for the 2 × 2 array of the unit structure.

**Figure 14:**
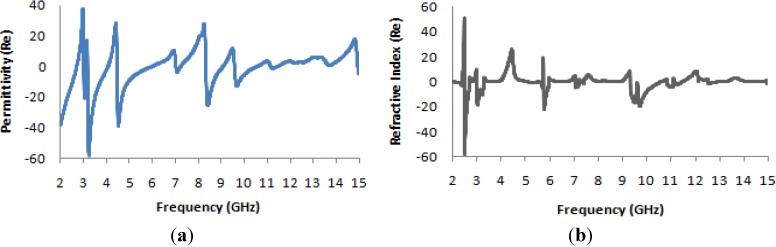
(**a**) The real value of permittivity *vs.* the frequency for the 2 × 2 array of the unit structure; (**b**) the real value of the refractive index *vs.* frequency for the 2 × 2 array of the unit structure.

It is evident from Figure 13a that the transmission coefficient exhibits resonance points that are slightly shifted from the resonance points of the unit cell, and it also exhibits some new resonance points (at 8 and 13 GHz); however, the resonances in the four microwave bands (S-band, C-band, X-band and K_u_-band) are maintained. The resonance points are most strongly shifted in the higher-frequency ranges. Figure 13b and Figure 14a present the real values of the effective permeability and permittivity *vs.* frequency for the 2 × 2 array of the unit structure.

In Figure 13b, it is apparent that the highest magnitude of the permeability, which occurs at a frequency very close to 5 GHz in all previously considered configurations, is shifted to a frequency of approximately 6 GHz; similarly, the maximum magnitude, which is very close to ±200 in all previously considered cases, has an absolute value of greater than 300 here. 

In Figure 14a, the permittivity characteristics of the 2 × 2 array of the unit structure as a function of frequency reveal that the maximum magnitude of the permittivity occurs very close to 3 GHz, as in the case of the 1 × 1 array of the unit structure, but the maximum magnitude remains within a range of +40 to −60, which are less extreme values than those observed for the permittivity curves of all previously considered structures of the unit cell. In Figure 14b, the refractive index also takes on negative values in the desired frequency bands, and Table 5 lists the corresponding values of the effective medium parameters.

In Table 5, there are a few notable differences from the previously considered structures. For example, the frequencies of interest at which double negativity occurs in the various microwave bands have changed slightly, particularly at higher frequencies. This structure exhibits a lower epsilon (ε) value at the lowest-frequency resonance (2.70 GHz) than all previously obtained experimental results for the unit cell. However, at the same (lowest) level of frequency, the effective permeability is higher than all previous results. This is the first time that the highest-value position of the refractive index has shifted toward 3 GHz from 2 GHz. 

**Table 5:** Real values of ε, μ and η in the desired resonance frequency bands for the 2 × 2 array of the unit structure.

Frequencies (GHz)	Value of ε	Value of μ	Value of η
2.70	−0.23	−25.47	−4.76
7.057	−3.074	−5.16	−4.64
10.5	−1.12	−3.65	−2.04
14.95	−1.37	−0.28	−1.10

### 4.4. Design and Analysis of a 2 × 2 the Unit Cell

**Figure 15:**
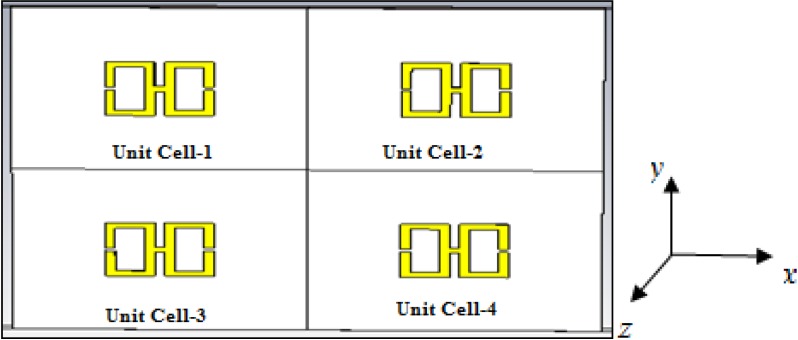
The 2 × 2 H-shaped unit cell.

As seen in Figure 15, in this structure, the entire unit cell is arranged in the same manner as the 2 × 2 array of the unit structure depicted in Figure 12. Operating frequencies between 2 and 15 GHz were considered, and the same methodology as used previously was used to evaluate the performance of the array.

**Figure 16:**
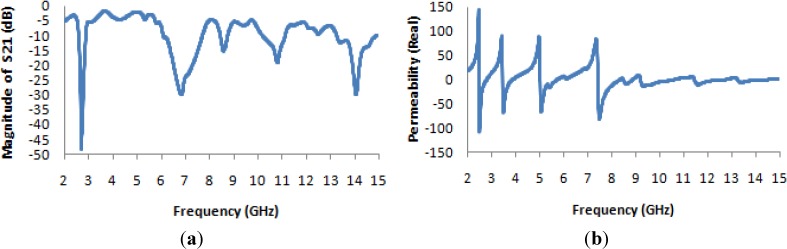
(**a**) The magnitude of the transmission parameter (S_21_) in units of dB for the 2 × 2 unit cell; (**b**) the real value of permeability *vs.* the frequency for the 2 × 2 unit cell.

Figure 16a presents the transmission coefficient and permeability for the 2 × 2 unit cell. From this figure, it is evident that the locations of the resonance points throughout the frequency range remain unchanged from the cases of the basic unit cell and the 1 × 1 unit cell, but that the magnitude is more negative at lower frequencies. The maximum magnitude of the permeability occurs between 2 and 3 GHz, as seen in Figure 16b.

**Figure 17:**
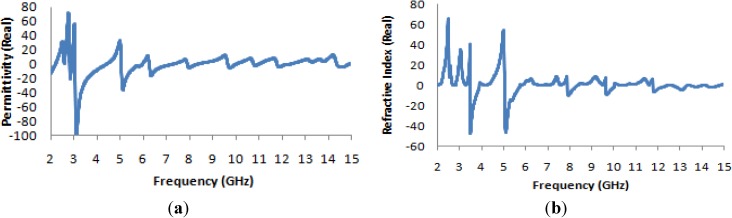
(**a**) The real value of permittivity *vs.* the frequency for the 2 × 2 unit cell; (**b**) the real value of the refractive index *vs.* frequency for the 2 × 2 unit cell.

The real value of the permittivity is presented in Figure 17a, where it is clearly apparent that the maximum value, which occurs near 5 GHz for the basic unit cell and the 1 × 1 unit cell, lies near 3 GHz for this structure. In Figure 17b, the refractive index is also seen to be negative in the desired frequency bands, as in the case of the original unit cell and the 1 × 1 unit cell array. 

In Table 6, which presents the values of the effective medium parameters, it is apparent that although the material exhibits the characteristics of a single-negative metamaterial in the lower frequency bands, it also exhibits a “zero refractive index”, which is another interesting area of study for researchers in the field of optical physics.

**Table 6:** Real values of ε, μ and η in the desired resonance frequency bands for the 2 × 2 unit cell.

Frequencies (GHz)	Value of ε	Value of μ	Value of η
2.74	66.52	−5.42	0.100
7.005	−2.165	23.85	0.13
10.84	−2.64	2.08	0.70
14.35	−0.46	−1.12	−2.34

A “zero refractive index” refers to a state of quasi-infinite phase velocity and infinite wavelength upon the entry of light. It also implies that if light enters a metamaterial with a zero refractive index, every point within the metamaterial experiences a quasi-uniform phase, as though all the dipoles inside the metamaterial were oscillating in harmony [34]. Thus, this phenomenon creates a uniform phase distribution, which has far-reaching consequences; for example, placing an emitter inside a zero-refractive-index material creates a radiation field of uniform phase inside the material, which can produce a highly directional beam at a planar surface [35]. However, in the K_u_-band (12–18 GHz) region, the material array still exhibits double negativity and a negative refractive index.

## 5. Comparative Analyses of the Configurations and Optimization Decisions

In the case of the S-parameter magnitude, the locations of the resonance points of the transmission parameter for both the single-unit cell structure and the 1 × 1 unit cell structure are nearly the same, but in the case of the 1 × 1 array structure in the same substrate plane, the magnitudes are more negative. This finding is attributable to the capacitive-loading effect of the presence of another parallel unit structure on the same substrate, which yields increased impedance.

**Figure 18:**
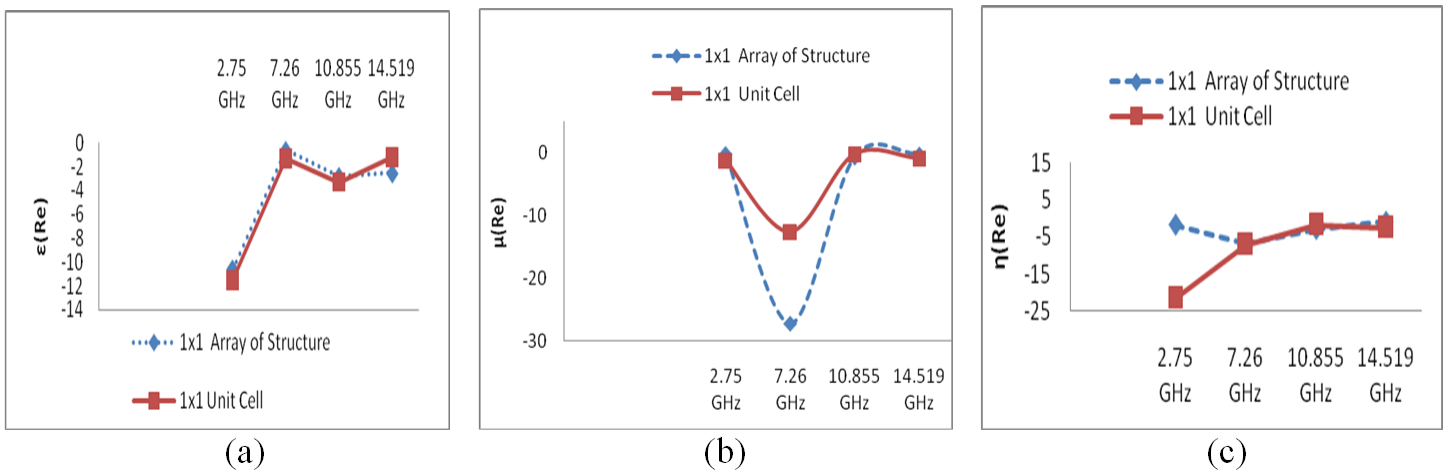
(**a**) ε-value comparison of 1 × 1 configurations; (**b**) μ-value comparison of 1 × 1 configurations; (**c**) η-value comparison of 1 × 1 configurations.

In Figure 18a, the comparison of the effective media parameters of the 1 × 1 array for both the unit structure and the 1 × 1 unit cell structure at frequencies of 2.75 GHz, 7.26 GHz, 10.85 GHz and 14.52 GHz reveals that the array of the unit structure exhibits nearly the same negativity as the 1 × 1 unit cell structure up to the X-band (8–12 GHz), but in the case of the K*_u_*-band, it exhibits increased negativity with respect to the 1 × 1 unit cell structure.

Figure 18b illustrates that the effective permeability for both 1 × 1 designs displays a near-zero value in all bands, except the C-band (4–8 GHz). Moreover, the same difference in negativity is also observed in the C-Band, as the 1 × 1 array structure exhibits more negativity than the 1 × 1 unit cell structure.

In the case of the refractive index, presented in Figure 18c, it is seen that the refractive indices of the 1 × 1 structures take nearly the same values in all bands, except in the lower S-band region, where the 1 × 1 unit cell structure displays more negativity than the 1 × 1 array of the unit structure. This discrepancy is attributable to the increased effective permittivity in this region.

In the case of the comparative study between the 2 × 2 array of the unit structure and the 2 × 2 unit cell structure, a comparison of the S-parameter magnitudes indicates that for the unit cell array, the transmission parameter exhibits the same resonance points as in the cases of the basic unit cell and the 1 × 1 unit cell structure, but the 2 × 2 unit structure exhibits two new resonance points at 8 and 13 GHz below −10 dB.

**Figure 19:**
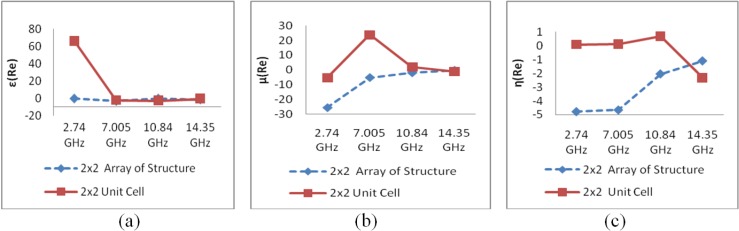
(**a**) ε-value comparison of 2 × 2 configurations; (**b**) μ-value comparison of 2 × 2 configurations; (**c**) η-value comparison of 2 × 2 configurations.

Figure 19a presents the comparison between the effective permittivity of the 2 × 2 array of the unit structure and that of the 2 × 2 unit cell structure at frequencies of 2.75 GHz, 7.05 GHz, 10.85 GHz and 14.35 GHz and demonstrates that with the exception of the lowest frequency (2.74 GHz) band, both configurations exhibit similar negativity with slight differences. However, at 2.75 GHz and 10.85 GHz, the 2 × 2 unit structure exhibits an epsilon near-zero (ENZ) value, which is a very interesting area of study for researchers in the field of high-directivity antennas [36].

In the case of the permeability, in Figure 19b, it is evident that the 2 × 2 array of the unit structure exhibits negativity in all four bands, whereas the unit cell structure exhibits negativity only at 2.75 GHz and 14.35 GHz. However, the magnitude varies considerably in the lower frequency bands (S- and C-bands).

In Figure 19c, it is seen that the refractive index for the 2 × 2 unit array structure exhibits negativity in all frequency bands under consideration, whereas the 2 × 2 unit cell structure displays a negative refractive index in the K_u_ band only. In other frequency bands, the refractive index of the 2 × 2 unit cell structure is zero or near zero.

As seen in Figure 18, another observation of interest is that the permittivity (ε) and refractive-index (η) curves for both the 1 × 1 array and the 1 × 1 unit cell configuration exhibit upward slopes toward zero at a different frequency from that of the permeability (μ) curve.

It is evident from Figure 19 that all of the curves of the effective medium parameters (ε, μ, and η) for the 2 × 2 unit array structure exhibit upward slopes with increasing frequency, whereas the curves of the effective parameters for the 2 × 2 unit cell structure exhibit downward slopes toward greater negativity with increasing frequency. Thus, the 2 × 2 array of the unit structure demonstrates better performance for double-negativity applications. On the other hand, the 2 × 2 unit cell structure demonstrates better performance for near-zero refraction index applications.

## 6. Conclusions

A new double-negative metamaterial unit cell structure is presented that resonates at frequencies in the S-band, C-band, X-band and K_u_-band of the microwave spectra. The unit cell structure is first proven to be a metamaterial; then, 1 × 1 and 2 × 2 configurations are analyzed for two different types of arrangements of the unit structure. It can be concluded from the analysis results that from the perspective of the transmission parameter, superior and more strictly maintained performance is achieved for the proposed 1 × 1 and 2 × 2 unit cell structures than for the proposed 1 × 1 and 2 × 2 array structures, but from the perspective of certain unique characteristics, it is observed that the 1 × 1 and 2 × 2 unit cells are superior for near-zero refractive index applications and that the 1 × 1 and 2 × 2 arrays of the unit structure are superior for double-negative applications. These structures can be used for multi-band applications and also for double-negativity or near-zero refractive index applications. Thus, these structures may be promising alternatives to other metamaterials, especially in applications that absolutely require metamaterials.
